# The interaction of the circadian and immune system: Desynchrony as a pathological outcome to traumatic brain injury

**DOI:** 10.1016/j.nbscr.2020.100058

**Published:** 2020-10-10

**Authors:** G.R. Yamakawa, R.D. Brady, M. Sun, S.J. McDonald, S.R. Shultz, R. Mychasiuk

**Affiliations:** a Department of Neuroscience, Central Clinical School, Monash University, Melbourne, Australia; b Department of Medicine, University of Melbourne, Parkville, Australia; c Department of Physiology, Anatomy and Microbiology, La Trobe University, Melbourne, Australia

**Keywords:** Concussion, Clock genes, Cytokines, Suprachiasmatic nucleus, Neuroinflammation

## Abstract

Traumatic brain injury (TBI) is a complex and costly worldwide phenomenon that can lead to many negative health outcomes including disrupted circadian function. There is a bidirectional relationship between the immune system and the circadian system, with mammalian coordination of physiological activities being controlled by the primary circadian pacemaker in the suprachiasmatic nucleus (SCN) of the hypothalamus. The SCN receives light information from the external environment and in turn synchronizes rhythms throughout the brain and body. The SCN is capable of endogenous self-sustained oscillatory activity through an intricate clock gene negative feedback loop. Following TBI, the response of the immune system can become prolonged and pathophysiological. This detrimental response not only occurs in the brain, but also within the periphery, where a leaky blood brain barrier can permit further infiltration of immune and inflammatory factors. The prolonged and pathological immune response that follows TBI can have deleterious effects on clock gene cycling and circadian function not only in the SCN, but also in other rhythmic areas throughout the body. This could bring about a state of circadian desynchrony where different rhythmic structures are no longer working together to promote optimal physiological function. There are many parallels between the negative symptomology associated with circadian desynchrony and TBI. This review discusses the significant contributions of an immune-disrupted circadian system on the negative symptomology following TBI. The implications of TBI symptomology as a disorder of circadian desynchrony are discussed.

## Highlights

• The immune system and circadian system interact with each other.
• During traumatic brain injury there can be a prolonged immune response.
• This immune response alters circadian rhythms in the body and brain.
• The body and brain may become desynchronized bringing about negative symptoms.

## 1 Introduction

Traumatic brain injury (TBI) is a complex worldwide phenomenon. Given that it is estimated that between 70 and 90% of all brain injuries are mild in severity (Dewan et al., 2018), and these types of injuries often go untreated, the true incidence of TBI is difficult to assess (Cassidy et al., 2004). Despite this, the healthcare costs of TBI have been estimated to be approximately 60 billion USD yearly (Coronado et al., 2015; Tardif et al., 2019). Particularly troublesome is the incidence of childhood or adolescent TBI, or multiple TBIs, which are commonly associated with sports, recreation, and drug or alcohol use, as this age group is undergoing critical brain development (Gogtay et al., 2004; Livingston et al., 2017). Estimates based upon all patients that have been hospitalized for TBI suggest that 43% go on to suffer from long term impairment (Selassie et al., 2013). While efforts have been made to describe the different severities of TBI in terms of mild, moderate, or severe, this review will primarily focus on impairments that are common following mild TBI (mTBI), although other severities will be discussed when necessary. Many of the acute impairments that follow mTBI resolve on their own, however for more than 30% of individuals with uncomplicated and even more with complicated mTBI, protracted post-concussive functional disturbances such as impaired memory, attention, fatigue, irritability, headache, and insomnia persist (Katz et al., 2015; Voormolen et al., 2019). More alarmingly, TBI may cause disruption to reward pathway maturation and subsequently lead to increased risk of later substance abuse disorders (Cannella et al., 2019). Other long term consequences include risk of epilepsy, stroke, cognitive deficits, emotional or temperamental change, increased risk of mortality, and early cognitive decline or dementia (Wilson et al., 2017; Brady et al., 2019).

**Fig. 1:**
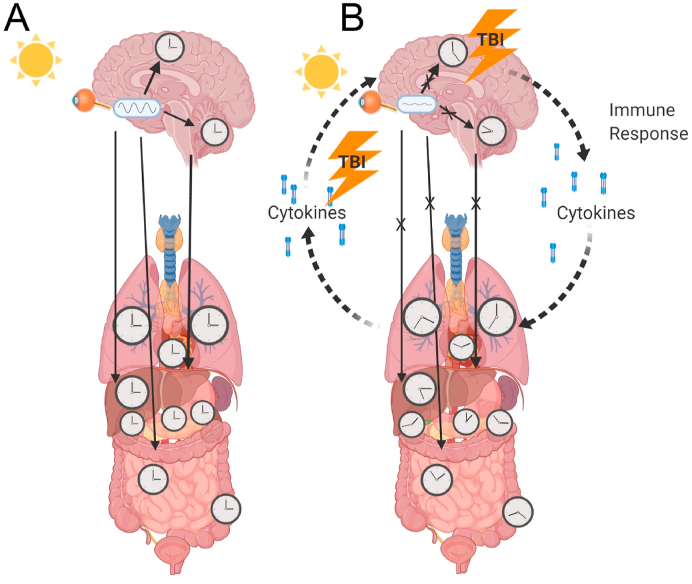
Hypothesized mechanism of circadian desynchronization after TBI. A: In an optimal normal state, the brain and periphery are synchronized to the external world through the SCN where rhythms are phase locked to occur at the appropriate times of the day or night. B: A pathological state that can be brought about by TBI, whereby there is a prolonged immune response that disrupts clock gene expression, bringing about a state of circadian desynchronization where there is disrupted signalling from the SCN, in addition to, disturbed rhythms in individual peripheral structures. A positive feedback in peripheral structures increases cytokine induced inflammation which can leak through the blood brain barrier and further exacerbate desynchrony. This loss of phase coherence among rhythmic structures could be a significant source of negative symptomology following TBI. Made with biorender.com.

Critical to the efforts of understanding the nature of TBI, particularly within the realm of mTBI, is to begin to appreciate and recognize the effects that these injuries have on natural biological rhythms and the pathophysiological responses that can result in long term detriment. Importantly, sleep disorders ranging from insomnia and fatigue, to narcolepsy occur in 30–70% of people that sustain a TBI (Viola-Saltzman and Watson, 2012). This suggests that a common consequence of TBI is a disruption of the circadian system. Coming from Latin for “Circa” or around, and “Diem” for a day, the circadian system is the biological machinery responsible for synchronizing physiological processes including sleep, to the solar day. Moreover, following TBI, there is a significant immune response that is essential for clearing out damage and initiating healing processes within the brain (Needham et al., 2019). There is also a bidirectional relationship between the circadian system and the immune system, where there are daily rhythms in immune function, and also the immune response can alter circadian function (Scheiermann et al., 2013; Hergenhan et al., 2020). Given this, we suggest that the circadian system can be disrupted by the immune system following a TBI. Notably, many reported consequences of a disrupted circadian system closely parallel the negative symptomology associated with TBI. The purpose of this review is to highlight the interactions between the immune and the circadian systems, while focusing on the pathophysiological responses of both to mTBI. If TBI is capable of producing prolonged and pronounced circadian desynchronization, and this can be linked to poor outcomes, important implications for the treatment of TBI will emerge. *We hypothesize that immune disruption of clock gene expression following TBI could lead to a state of circadian desynchronization that contributes to the presentation of post TBI symptoms.* An illustrative overview of the focus and primary objectives of this review article can be found in Fig. 1.

## 2 Core components of the circadian clock and the immune system

The mammalian primary circadian clock is located in the SCN of the anterior hypothalamus (Stephan and Zucker, 1972). The SCN receives light information along the retinohypothalamic tract which in turn synchronizes endogenously rhythmic cells and drives a transcription translation feedback loop of clock gene expression (Antle and Silver, 2005). This core molecular feedback loop involves the expression of Circadian Locomotor Output Cycles Kaput (*Clock*) and Brain and Muscle Arnt-like protein-1 (*Bmal1*), which heterodimerize and subsequently bind to the promoter regions of clock controlled genes, including Period 1–3 (*Per*) and Cryptochromes 1 and 2 (*Cry*) (Buhr and Takahashi, 2013). PER and CRY are expressed and translocate back into the nucleus where they halt the expression of *Clock* and *Bmal1* in a negative feedback loop that takes approximately 24 h to complete (Reppert and Weaver, 2001). There are also accessory feedback loops that stabilize this core loop involving negative regulation of *Bmal1* by *Rev-Erb Α* and the promotion of degradation of PER by Casein Kinase 1 (Cho et al., 2012; Zheng et al., 2014). Light input, in addition to behavioural and physiological stimuli such as food intake, activity, and body temperature, converge to produce stable synchronization or entrainment of mammals (West and Bechtold, 2015).

The SCN has been conceptualized as the principal circadian clock as it exerts phase and period control over other rhythmic areas, or subordinate oscillators not only in other regions of the brain, but also in the periphery (Guilding and Piggins, 2007). The SCN utilizes a combination of direct neural projections to areas such as the dorsal medial hypothalamus, arcuate, subparaventricular zone, and paraventricular nucleus (PVN), and circulating diffusible factors in order to communicate time information to the rest of the brain and body (Silver et al., 1996; Dibner et al., 2010). Experimental evidence for this has been shown when lesions of the SCN were made in PER2:Luciferase reporter mice and a loss of phase coherence among peripheral tissues was identified (Yoo et al., 2004). Additionally, using a *Per1* luciferase transgenic rat line, areas of the brain were isolated and cultured, with over half of the areas examined exhibiting rhythmicity that quickly dampened when connectivity to the SCN was removed, demonstrating that SCN function has widespread downstream effects across much of the brain (Abe et al., 2002). Interestingly, recent evidence has emerged to suggest that the liver, kidneys, and gastrointestinal system can function as at least semi-autonomous oscillators, albeit with a lower amplitude, in the absence of the SCN (Guilding and Piggins, 2007; Tahara et al., 2012; Koronowski et al., 2019). This has led to the conception of the SCN as an orchestra conductor, helping to keep time and maintain synchrony between each of the constituent parts (Antle and Silver, 2005).

As previously mentioned, a bidirectional relationship between the circadian system and the immune system is well established. The brain is able to respond to an immune challenge through the hypothalamus to bring about physiological adaptations, or the induction of sickness type behaviour (Soto-Tinoco et al., 2016). Pro-inflammatory cytokines can increase sleep, reduce circadian output, phase shift circadian rhythms, and alter photic entrainment (Cermakian et al., 2013; Labrecque and Cermakian, 2015). The likely interface between the immune system and the SCN is astrocytes. The SCN shows dense staining for glial fibrillary acidic protein (GFAP), a marker of astrocytes and these cells exhibit immune related expression of transcription factor kB (NF-kB), which has been shown to be activated in the presence of lipopolysaccharide or TNFα (Leone et al., 2006). Other possible immune circadian interfaces include glucocorticoids, melatonin, leptin, and prostaglandins, all of which have been suggested to play roles in communicating immune information to the circadian system (Cermakian et al., 2014).

Given immune factors can influence circadian function, the SCN can then coordinate and influence the immune responses through the PVN, endocrine, and autonomic neurons (Logan and Sarkar, 2012). Microglia isolated from older rats showed aberrant PER, TNFα, and interleukin-1β (IL-1β) rhythms, as well as, entrainment signals indicating that the circadian system influences the immune system and this control is lost with aging (Fonken et al., 2016; Sun et al., 2020). Components of the immune system that have been shown to have circadian variation include neutrophils (Aroca-Crevillen et al., 2020), leukocytes (Pick et al., 2019), macrophages (Martinez-Tapia et al., 2020), lymphocytes (Arjona and Sarkar, 2005), microglia (Fonken et al., 2015), and monocytes (Nguyen et al., 2013). Large shifts of the light-dark cycle or shift work paradigms that created a circadian misalignment brought about a 4-fold increase in mortality and an exaggerated cytokine inflammatory response following experimental immune challenge, while also producing alterations or complete loss of clock gene rhythms in the SCN, liver, and macrophages also showing the strong influence of the circadian system on the immune system (Castanon-Cervantes et al., 2010; Guerrero-Vargas et al., 2015).

## 3 Cytokines in the sleep and circadian system

Given that pro-inflammatory cytokines can influence circadian activity, this is another aspect of immune function that has received significant interest in the literature. Exposure of stem cell derived neuronal cultures to TNFα, IL-6 and IL1β levels typical of TBI resulted in dose and time dependent cytokine release (Thelin et al., 2018). TNFα has multiple functions including apoptosis, inflammation, promotion of calcium homeostasis, and negative regulation of melatonin and immune responses (Ertosun et al., 2019). There are two known receptors for TNFα that exhibit widespread expression throughout the central and peripheral nervous system. The P55 or TNFR1 receptor is ubiquitously expressed at low levels in ependymal walls, the arcuate, supraoptic nucleus, and cerebellum (Nadeau and Rivest, 1999). Conversely, the P75 or TNFR2 neurotrophin receptor oscillates in a circadian manner due to binding of the CLOCK/BMAL1 heterodimer within the Ebox promoter region (Baeza-Raja et al., 2013). Importantly, both receptors have been detected in the SCN, and *in vitro* application of TNFα caused a nitric oxide dependent increases in neuronal firing rates (Nygard et al., 2009). Another study found that media taken from cultured SCN astrocytes treated with TNFα could phase shift SCN PER2 rhythms (Duhart et al., 2013). In addition, TNFα can halt the dimerization of CLOCK and BMAL by binding to the EBox promoters of PER (Cavadini et al., 2007). TNFα has also been shown to increase AMPA receptor expression thereby strengthening glutamate signalling (Beattie et al., 2002). Importantly, the SCN communicates with peripheral oscillators through the PVN, supraoptic nucleus, and arcuate (Vrang et al., 1995; Saeb-Parsy et al., 2000). TNFα receptors are also present in the PVN where they can modify post-synaptic signalling which suggests that not only can TNFα modify direct activity at the SCN, but also SCN output to peripheral oscillators (Glass et al., 2017).

Another pro-inflammatory cytokine, interferon gamma (IFNγ), acts in a manner similar to TNFα, whereby it decreases excitatory activity in SCN cultures while also suppressing PER1 rhythmic expression amplitude (Kwak et al., 2008). The interleukins are another group of cytokines involved in the immune response. IL1α and IL1β can both bind to the IL-1 receptor 1 (IL-1R1) (Thome et al., 2019). There is evidence that IL1β may also act in a manner similar to TNFα, as they both increase non-rapid eye movement sleep, and bring about sickness behaviours such as lethargy, weakness, and altered sleep wake cycles (Coogan and Wyse, 2008). Both IL1β and IL-1R1 are expressed in the SCN of mice, with IL-1R1 showing diurnal variation, and upregulation in response to immune challenge (Beynon and Coogan, 2010). IL6 is another pro-inflammatory cytokine that increases with age, or in response to stress and other inflammatory states that is an important driver of the propensity to sleep (Rohleder et al., 2012). IL6 has been shown to increase in peripheral organs *in vitro* in response to environmental and immune challenge, while increased release promotes sleepiness (Vgontzas et al., 1999; Stowie et al., 2019). Importantly, IL6 is capable of activating signal transducers and activators of transcription proteins, and these proteins are expressed in the SCN, which provides a direct interface between IL-6 and the circadian system (Moravcova et al., 2016; Verboogen et al., 2019).

There is considerable evidence that cytokines can directly influence sleep. TNFα and IL1 can suppress activity of orexin neurons and are associated with sickness type behaviour including increased sleep time and reduced appetite (Clark and Vissel, 2014). Experimental diffuse brain injury induced an acute increase in cytokines and increased sleep more in male mice when compared to females; whereas females experienced more peripheral inflammation (Saber et al., 2020). TNFα release acutely produces an inflammatory brain responses that is likely responsible for the increases in sleep seen post-TBI (Rowe et al., 2018). Brainstem regions that have been associated with sleep disturbances have also been found to be suppressed, show increased cytokine release, gliosis, and even exhibit cell loss after mTBI, all of which are potential mechanisms by which cytokines influence sleep (Sinha et al., 2017). For example, patients suffering from obstructive sleep apnea show higher levels of IL-6 (Motamedi et al., 2018), while veteran populations exhibit significant correlations between blood TNF markers, fatigue, and sleeping difficulties (Cheng et al., 2020).

## 4 The immune system response following TBI

TBIs are associated with initial biomechanical linear and rotational forces that may result in widespread white matter damage throughout the brain (Bigler, 2001; Buki and Povlishock, 2006). This immediate damage results in ionic influx and efflux, glutamate release, increases in mitochondrial energy demands, altered neurotransmission, and neuroinflammation (Giza and Hovda, 2014). This neuroinflammation includes activation of glial cells and leukocytes, and production of cytokines in an effort to clear out cellular debris and promote restorative processes (Xiong et al., 2018). Damage associated molecular patterns such as reactive oxygen species produced with the initial injury promote activation of the NLRP3 inflammasome which promotes IL1β and IL18 release (O'Brien et al., 2020). This NLRP3 expression is under circadian control and when there are disruptions to the circadian system, there is also increased inflammation (Pourcet and Duez, 2020).

Immunohistochemical markers show increased glial cell activation post-TBI (Pham et al., 2019). The glial cell activation can also result in a significant release of pro-inflammatory cytokines including TNFα, IL1β, and IL6 (Singh et al., 2016). Excessive and prolonged levels of pro-inflammatory cytokines can act on glial cells to negatively regulate neurogenesis and also result in further release of cytokines, as a self-renewing process (Xiong et al., 2009; Bertini et al., 2010). In an investigation of cognitive deficits following mTBI, patients that presented with higher acute levels of serum cytokines showed worse cognitive symptoms (Sun et al., 2019b). Repeated mild stretch injuries act on cultured astrocytes to increase susceptibility to IL1β and increased matrix metalloproteinase-9 expression, which may serve as an underlying mechanism for increased susceptibility to poor outcomes with successive insults (Ralay Ranaivo et al., 2011). Moreover, data collected in a longitudinal survey revealed that patients with a prior history of concussion showed longer subsequent concussion duration, higher reported sleep disturbances, headaches, and cognitive difficulties (Oyegbile et al., 2019).

In addition to the brain's release of cytokines, there is also a peripheral response where the spleen, thymus, and bone marrow release cytokines (McDonald et al., 2020). Through a leaky blood brain barrier post TBI, peripheral cytokines may also contribute to neuroinflammatory processes (Das et al., 2012) There are high levels of TNFα and IL6 released in the small intestine, that remain elevated for at least one week following TBI (Hang et al., 2005). In addition, following TBI, circulating cytokines accumulate in the liver, which results in organ inflammation and the release of chemokines which can lead to further inflammation of other organs (Moser and Willimann, 2004; Villapol, 2016). TNFα and IL1β were found to suppress *Per1-3* and the SCN controlled gene D-site binding protein (*DBP*) (Cavadini et al., 2007). DBP works in concert with CLOCK/BMAL to activate the E-Box promoter of *Per*. Since the peripheral organs including the liver, small intestine, large intestine, stomach, heart, lungs, and kidneys all show rhythms in clock gene expression, the brain's immune response leads to a peripheral inflammatory response, and this inflammation can further suppress core clock genes. Therefore, TBI results in a state of circadian desynchrony, where not only is there disruption of entrainment signalling from the SCN, but also a disruption of individual rhythms in central and peripheral oscillatory structures.

## 5 Circadian desynchrony and TBI symptomology

For some time, there has been a growing interest in the influence of circadian rhythms on health problems and psychiatric conditions. A systematic analysis of RNA in mice demonstrated circadian expression of ~43% of protein coding RNA, and more than a 1000 novel non-coding RNAs that oscillated in a circadian manner (Zhang et al., 2014). When there is a loss of coherence in circadian rhythms, physiological parameters occur at non-optimal times or are suppressed completely. For example, food intake during a time when intestinal rhythms of nutrient absorption are low has been shown to result in greater obesity, insulin resistance, and hypertension, suggesting that circadian rhythms optimize the body to prepare for food at particular times of the day (Zimberg et al., 2012). Additionally, a loss, or change in gut microbiota that comes with circadian misalignment may also produce metabolic alterations or inadequate absorption of nutrients (Parkar et al., 2019). Hamsters made arrhythmic showed profound memory deficits that were reversed by lesioning the SCN, indicating that an abnormal signal from the SCN was more detrimental than no signal (Fernandez et al., 2014). Prolonged circadian desynchronization, or a lack of internal and or external synchrony between rhythmic physiological functions has been shown to elevate rates of cancer, cardiovascular, metabolic, and psychiatric disorders (Touitou et al., 2017). Large phase shifts of the light-dark cycle require not only the SCN neurons to resynchronize but also peripheral oscillators to resynchronize to each other and the SCN, in a process that can take weeks (Krishnan and Lyons, 2015). To date, circadian desynchrony brought about by the immune response following TBI remains understudied. The numerous documented effects of internal desynchronization are often analogous to post-TBI symptomology (West and Bechtold, 2015). Symptoms resulting from circadian disruption experimentally induced through jet lag or shift work, along with the symptoms commonly experienced after TBI are displayed in Table 1 and discussed in the following sections.

**Table 1:** Impairments common to Post-TBI and circadian desynchronization.

	Post TBI Symptomology	Circadian Desynchronization
**Cognitive Deficits**		

**Attention**	(Cicerone, 1996; Halterman et al., 2006; Pare et al., 2009; Bolduc-Teasdale et al., 2019)	(Maire et al., 2018; Valdez, 2019; McGowan et al., 2020; Smit et al., 2020)
**Memory**	(Arciniegas et al., 2002; Eyolfson et al., 2020; Macheda et al., 2020)	(Cho et al., 2000; Craig and McDonald, 2008; Sakamoto et al., 2020)
**Executive Function**	(Brooks et al., 1999; Rabinowitz and Levin, 2014; Kunker et al., 2020)	(Walsh et al., 2014; Kuula et al., 2018; Choshen-Hillel et al., 2020)

**Mood Disorders**		

**Depression**	(Walker et al., 2015; Ouellet et al., 2018; Frankot et al., 2020; Papadaki et al., 2020)	(Asarnow et al., 2013; Albrecht, 2019; Khairuddin et al., 2020; Pandi-Perumal et al., 2020)
**Anxiety**	(Barker-Collo et al., 2018; Gillie et al., 2020; Hsu et al., 2020; Lecuyer Giguere et al., 2020)	(Griesauer et al., 2014; Coles et al., 2015; Kirlioglu and Balcioglu, 2020)
**Disrupted HPA Axis**	(Javed et al., 2015; Kosari-Nasab et al., 2019b; Tapp et al., 2019)	(Nader et al., 2010; Nicolaides et al., 2014; Dumbell et al., 2016)

**Physical Impairments**		

**Sleep**	(Barshikar and Bell, 2017; Brustman et al., 2020; Iyer et al., 2020)	(Flynn-Evans et al., 2017; McHill et al., 2018, 2019)
**Intestinal Permeability**	(Bansal et al., 2009; Zhu et al., 2009; Duan et al., 2013; Konig et al., 2016)	(Pan and Hussain, 2009; Summa et al., 2013; Swanson et al., 2020)
**Cancer**	(Chen et al., 2012; Tyagi et al., 2016; Juskys and Chomanskis, 2020)	(Feillet et al., 2015; Padmanabhan and Billaud, 2017; Vitale et al., 2018)
**Cardiovascular**	(Hilz et al., 2015; Ramos-Cejudo et al., 2018; Johnson et al., 2020; Sercy et al., 2020)	(Khaper et al., 2018; Chalfant et al., 2020; Mia et al., 2020)
**Insulin Resistance**	(Karelina et al., 2016; Li and Sirko, 2018; Franklin et al., 2019; Pomytkin et al., 2019)	(Zhong et al., 2019; Toyoura et al., 2020; Wang et al., 2020; Yin et al., 2020)
**Dysbiosis**	(Treangen et al., 2018; Angoa-Perez et al., 2020; Bowers et al., 2020)	(Voigt et al., 2016; Hong et al., 2020; Liu et al., 2020; Mashaqi and Gozal, 2020; Matenchuk et al., 2020)

For each symptom listed in the leftmost column, we have cited examples of research showing that these are presented both in post-TBI and in cases of circadian desynchronization. For ease of organization, the symptoms are divided into cognitive deficits, mood disorders and physical impairments. We have included references in the table that are not described in the text in an effort to highlight the extensive overlap and direct the reader to additional literature on this important topic.

### 5.1 Sleep

Some of the most commonly reported symptoms associated with circadian desynchronization and TBI are insomnia and sleeping difficulties (Mathias and Alvaro, 2012). A study of 87 patients, assessed at 3 months following TBI, found that 46% had sleep disorders (Castriotta et al., 2007) and sleep disturbances have been shown to impair recovery (Fleming et al., 2020). In addition, a high incidence of circadian rhythm disorders that are often mischaracterized as insomnias, parasomnias, and dyssomnias, where the individual experiences difficulties falling asleep, have also been associated with TBI and these circadian disorders have been shown to increase recovery time (Mazwi et al., 2015). The SCN controls sleep-wake behaviours through consolidation of behavioural states, which promote arousal during the wake period (Easton et al., 2004). Therefore, sleeping problems following TBI may result from circadian disruption. For example, we have previously shown that RmTBI causes reductions in vasoactive intestinal polypeptide (VIP) expression in the SCN (Yamakawa et al., 2019). VIP cells in the SCN receive light information from the retinohypothalamic tract and exert a powerful synchronizing effect on the rest of the SCN (Hamnett et al., 2019). The coup-contrecoup injury associated with TBI may also affect the basal forebrain arousal area, damage the SCN, interrupt melatonin pathways, or disrupt thalamocortical connectivity, all of which have the potential to contribute to post-traumatic sleeping difficulties (Zhou and Greenwald, 2018). Previous work has shown that the basal forebrain is involved in direct regulation of circadian rhythms in the SCN (Yamakawa et al., 2016).

More severe injuries have resulted in increased incidence of daytime sleepiness which has been associated with symptom severity and anxiety measures (Crichton et al., 2020). A sample of TBI patients showed significantly longer sleep times and daytime sleepiness than healthy controls, which persisted at least 18 months following injury (Imbach et al., 2016). Older male adults in particular appear to show more sleeping difficulties following TBI, including apnea and daytime sleepiness, which contributes to cognitive deficits (Wei et al., 2020). TBI-induced sleeping difficulties have be associated with damage to sleep wake circuits including the VLPO, orexinergic, histaminergic, and serotonergic systems (Werner and Baumann, 2017). Autopsied brain tissue from patients with severe TBI showed significant loss of hypothalamic orexin neurons (Baumann et al., 2009). A separate post-mortem examination revealed an increase in hypothalamic gliosis and a significant loss of histaminergic and melanin concentrating hormone containing neurons which could contribute to daytime sleepiness and disturbed rapid eye movement sleep (Valko et al., 2015).

A study of females presenting with insomnia found that a significant proportion were trying to commence sleep at a time earlier than their melatonin onset, suggesting circadian disruption was contributing to the disorder (Flynn-Evans et al., 2017). Further, it has been suggested that insomnia is indicative of a hyperarousal. Global metabolic profiles were different in insomnia patients where, particularly at night, increases in glucose, amino acids, and energy metabolites were identified, suggesting that peripheral desynchrony was contributing to the sleeping difficulties (Gehrman et al., 2018). It has been suggested that the circadian system requires feedback from the periphery in order to synchronize processes, thus a disruption of peripheral oscillations could also bring about desynchronization of the network (Buijs et al., 2016). Insomnia patients have been found to have higher, more variable heart rates, increased cortisol secretions, elevated body temperature, and metabolic rates, all of which suggest peripheral desynchrony (Bonnet and Arand, 2010). There is a rapid response from the liver to release cytokines in response to brain injury increasing peripheral inflammation and interfering with clock gene function (Villapol, 2016). This peripheral desynchronization interacting with SCN dysregulation could be a major contributing factor to the development of sleep disorders.

As a normal response to infection, the immune system has been shown to be able to directly interact with the brain through cytokine release to bring about sickness type behaviours (Dantzer, 2009). Specifically, more frequent rest periods and a decrease in locomotor activity were seen after TNFα exposure likely occurring due to the suppression of *Per* and *Dbp* (Cavadini et al., 2007; Coogan and Wyse, 2008). IL1β is known to activate the ventrolateral preoptic area, a major brain region involved in the initiation of sleep (Baker et al., 2005). Blocking either TNFα or IL1β decrease sleep, while levels of these cytokines significantly increase in response to sleep deprivation (Krueger et al., 2011). A review of the literature suggests that sleep disorders have multiple outcomes and trajectories following injury and may actually impair recovery and treatments. Further, therapies that focus on sleep hygiene and light therapy have had some success in treating TBI-induced sleep disorders (Barshikar and Bell, 2017). One particular study found that insomnia and depression post-TBI was associated with the fatigue caused by diffuse axonal injury and impairments in information processing (Tomar et al., 2018). The high incidence of reported sleep problems following TBI could therefore be a result of circadian desynchrony brought about by damage to SCN input pathways, or immune system changes to clock gene expression. This may provide an important avenue for treatment.

### 5.2 Cognitive processing

Following TBI, cognitive impairment is frequently reported and is a significant source of negative symptomology. Experimental forced desynchrony protocols have shown that there are circadian rhythms in cognitive performance in addition to the generally worse cognitive performance seen as a function of tiredness, or sleep drive (Burke et al., 2015; Valdez, 2019). Both basic cognitive processes such as attention and more complex processes such as executive function, social behaviour, and motivation can be disrupted as higher order functions depend on basic processes (Arciniegas et al., 2002). In an animal model of jet lag, phase advances of the light-dark cycle produced reductions in hippocampal neurogenesis and long-term impairments in hippocampal dependent memory that persisted even after the animal was returned to a normal light-dark cycle (Gibson et al., 2010). Chronic circadian desynchrony brought about by repeated transmeridian travel in airline crews has been shown to produce increased cortisol levels as well as persistent memory and cognitive deficits (Cho et al., 2000). Since both diurnal and nocturnal animals show peak memory performance during their active periods despite having similar rhythms of SCN clock gene expression, circadian cognitive performance deficits are likely downstream, and may rely on hippocampal gene expression or melatonin rhythms (Krishnan and Lyons, 2015). Rats placed in a chronic state of desynchrony showed deficits in hippocampal spatial memory further suggesting that aberrant SCN signalling disrupts hippocampal rhythmicity and manifests in memory deficits (Craig and McDonald, 2008).

TBI patients have been shown to suffer from deficits in working memory, long-term memory, executive function, social skills, and information processing delays (Azouvi et al., 2017). For example, when mTBI patients were asked to perform a cognitive task in the presence of a distraction they showed worse processing speed (Cicerone, 1996). Pre-clinical studies have provided evidence that the immune response may be a key factor to cognitive deficits after TBI It has been demonstrated that an immune challenge following TBI significantly exacerbated cognitive deficits and greatly increased the glial inflammatory response (Muccigrosso et al., 2016). Depletion of chronically activated microglia one month after TBI was found to reduce lesion size, cell death, neuroinflammation, and improve cognitive outcome in mice (Henry et al., 2020). A pathological immune response to TBI likely induces a state of chronic circadian desynchrony. Prolonged inflammatory responses have been found to negatively correlate with cognitive function and also are a risk factor for chronic disease (Sartori et al., 2012). Rhythms in the hippocampus are normally dependent on SCN driven rhythms of corticosterone release and loss of this rhythm has been found to abolish hippocampal dependent learning processes (Snider et al., 2018). Thus, a prolonged state of neuroinflammation, leading to a state of circadian desynchronization, could be contributing to cognitive difficulties following TBI.

### 5.3 Cancer

The association between TBI and cancer remains mixed. Since cell division is under circadian control, and SCN lesioned mice show a higher incidence of cancer, it can be argued that cancer is at least partly a disorder of disrupted circadian rhythms (Lamont et al., 2007). Molecular work has shown that prolonged disruption of the circadian clock enhances the proliferation phase of the cell cycle (Lee et al., 2019). Single cell imaging in combination with a computational biology approach, suggests the circadian system slows and synchronizes the cell cycle and during circadian desynchrony, this process becomes maladaptive resulting in greater tumour formation (Feillet et al., 2015). Experimental manipulations that induced clock gene expression slowed tumour growth in cultured melanoma and carcinoma cells (Kiessling et al., 2017). Shift work has been classified as a probable carcinogen with exposure to bright light during the night and disrupted sleeping schedules creating a perpetual state of circadian misalignment (Savvidis and Koutsilieris, 2012). Circadian transcriptome analysis has revealed with those transcripts that change with mistimed sleep were also those that were most shown to interact with cancer (Archer and Oster, 2015).

Earlier studies and some reviews revealed no relationship between previous head trauma and incidence of brain tumours (Annegers et al., 1979; Bondy and Ligon, 1996), however other studies revealed small associations and increased odds of developing brain tumours, particularly in males (Preston-Martin et al., 1983; Inskip et al., 1998). Surprisingly, recent large population-based samples revealed a significant association and greater incidence between sustaining a TBI and later developing brain or systemic cancers (Chen et al., 2012; Wee et al., 2019). A prolonged inflammatory state, not only affecting SCN signalling, but also clock gene rhythms in individual structures would remove the circadian slowing of the cell cycle throughout the brain and body resulting in greater time spent in the proliferation phase (Feillet et al., 2015). Conceivably, a loss of circadian synchrony following sustained immune response after TBI could remove control over the cell cycle and result in enhanced cell proliferation and subsequent tumour growth.

### 5.4 Psychological conditions

A considerable source of negative outcomes following TBI include reported psychiatric disturbances. These can include depression, mania, post-traumatic stress disorder, alcohol or substance abuse, and personality changes (Schwarzbold et al., 2008). There is also substantial overlap between the incidence of psychiatric conditions in TBI patients and both experimental and epidemiological studies of individuals suffering from circadian desynchrony. Interestingly, there is a proposed bidirectional relationship between mood disorders and circadian rhythms. Circadian disruption can exacerbate symptoms in those suffering from mood disorders and those that suffer from psychiatric disorders show disrupted circadian rhythms (Walker et al., 2020). In an experimental model of forced desynchronization, rats displayed a depressive phenotype including increased time spent immobile in the forced swim, sexual dysfunction, and reduced preference for saccharin (Ben-Hamo et al., 2016). It has been suggested that depression results from a complex interaction between genes, disrupted circadian rhythms, altered neurotransmission, psychological, and social factors (Salgado-Delgado et al., 2011). Sleep and circadian disruptions have also been reported in schizophrenia, anxiety, and bipolar disorder, with treatments that target the circadian system having some success in alleviating symptomology (Asarnow et al., 2013; Coles et al., 2015). Variations on the human *Per3* gene have been shown to manifest in higher rates of anxiety and mathematical models predict that this may occur through changes in binding to CRY or casein kinase (Liberman et al., 2017). Interestingly, mice that were bred to express high anxiety-like behaviour also show a reduction in *Cry2* expression in the hippocampus, in addition to altered phase shifting, period, and fragmented ultradian rhythms, suggesting abnormal circadian function (Griesauer et al., 2014).

Depressive and anxiety symptoms were found to be comorbid in about 10–20% of mTBI patients (Barker-Collo et al., 2018). A study found a higher prevalence of major depression following mTBI, than in response to moderate or severe injuries, with approximately 1/3 of patients with minor depression deteriorating to experience a major bout (Ouellet et al., 2018). Female patients with pre-existing depressive symptoms who are unemployed and suffered a loss of brain tissue during TBI appear to be at particular risk for developing major depression (Cnossen et al., 2017). Especially prevalent is the development of anxiety-like symptoms in military personnel following blast injury (Hoge et al., 2008), with preclinical models recapitulating this increase in anxiety and postulate that it may be mediated by direct changes to the limbic system (Russell et al., 2018). Notably, circadian mechanisms are involved when the limbic system timestamps fear memories and may be involved in fear conditioning (Albrecht and Stork, 2017). Animal studies utilizing RmTBI models show increased anxiety-like behaviour in the elevated plus maze and open field (Broussard et al., 2018), as well as increased depressive-like behaviours in the forced swim test (Salberg et al., 2017, 2018) that were associated with neuroinflammation. There is an increase in corticosterone and adrenocorticotropic hormone suggesting that the hypothalamic pituitary adrenal axis is hyperactive in response to TBI (Kosari-Nasab et al., 2019a). This increase in stress signalling may be a result of alterations of glucocorticoid receptor expression which is modulated by CRY and REV-ERBα (Albrecht, 2019). Glucocorticoid release is an important synchronizing signal of the circadian pacemaker relying on the SCN and rhythmicity within the adrenal glands themselves (Dickmeis, 2009). While being the result of circadian desynchronization and TBI, depression has also been conceptualized as immune disorder. Cytokines and glucocorticoids are elevated in the brains of depressed individuals which causes a reduction in serotonin synthesis and increased apoptosis (Leonard, 2010). Finally, the immune system has also been implicated in the development of obsessive compulsive disorder (Marazziti et al., 2018), as a potential mechanism for the development of substance abuse disorders (Montesinos et al., 2016), and in the development of anxiety disorders (Leonard and Song, 1996). These findings suggest that a prolonged immune response following TBI could be significantly altering circadian function enough to contribute to the development of post-traumatic psychological conditions.

### 5.5 Gastrointestinal/metabolic dysfunction

Another consequence of circadian misalignment has been poor coordination of metabolism and the absorption of nutrients. The intestine has been found to exhibit robust rhythmicity and clock gene expression for intestinal absorption of nutrients occurs at a phase similar to the liver, but delayed from the SCN by ~6 h (Balakrishnan et al., 2012). Mutations in the *Clock* gene were found to disrupt the diversity of gut microbiota (Voigt et al., 2016). Levels of tight junction proteins and mRNA show circadian cycling, while homozygous Per2 mutant mice exhibit constitutively high tight junction protein expression and consistently low intestinal permeability (Kyoko et al., 2014). In addition, Clock mutant mice show altered absorption of nutrients, presumably through changes in intestinal transport protein regulation (Pan and Hussain, 2009). Circadian dysfunction therefore impairs gastrointestinal function. Immune disruption of the circadian system following TBI could accordingly also contribute to these negative gastrointestinal symptoms. Metabolomics profiling of mice on standard chow versus a high fat diet, showed a loss of temporal synchronization of metabolites across and within different tissues when challenged with the high fat diet (Dyar et al., 2018). Circadian desynchrony induced by constant lighting conditions also had an effect on gut microbiota, clock genes, and antioxidant activity (Klimina et al., 2019).

Dysautonomia can occur following TBI where there is a loss of normal sympathetic and parasympathetic function which leads to inability to properly coordinate the digestion of food, increased intestinal permeability, increased expression of TNFα, damage to intestinal mucosa, and decreased intestinal contractile ability (Katzenberger et al., 2015; Kharrazian, 2015). Consistent with this, following RmTBI, rat jejenum showed significantly less bacterial diversity as early as 6 h post-injury (Matharu et al., 2019). These changes may have resulted from TBI induced modifications to the expression of tight junction proteins in the ileum, which were significantly reduced (Bansal et al., 2009), and are under circadian control (Kyoko et al., 2014). Further, experimental TBI has been shown to produce chronic pathological changes in the colon, with further bacterial infections exacerbating not only gut dysfunction, but also TBI induced brain neuropathology (Ma et al., 2017). TNFα appears to play an important role in intestinal permeability, as it has been shown to decrease intestinal tight junction levels and potentiate inflammation, bringing about intestinal cell death (Patterson et al., 2019). The immune response following TBI could therefore doubly affect GI function by directly affecting clock gene expression in the SCN and periphery bringing about a state of circadian desynchrony, but also by disrupting the function of the GI system itself.

## 6 Conclusion and future directions

The consequences and symptoms of TBI are diverse. We are just beginning to understand the long-term consequences of TBI and the recovery trajectories for post-traumatic symptomologies. This review highlighted the importance of understanding TBI as a factor that contributes to and may disrupt the complex interaction between the immune and circadian systems. Increasing our understanding of the circadian and immune system response to injury can help to elucidate the complex outcomes of TBI and may shed light on more innovative treatments. For example, recent work in inflammation has found mouse cortical cytokines and chemokines show temporal expression pattern changes from as soon as 8 h following a single closed head injury to as long as 30 days post post-TBI (Tweedie et al., 2020). Further characterization of the specific impairments to the circadian system can also advance understanding of TBI outcomes and help to better inform treatment. A recent preclinical study found decreases in vasopressin, another important circadian neurotransmitter, in specific brain regions that showed morphological deficits (Hosgorler et al., 2020). Therefore, future studies may demonstrate that restoration of vasopressin levels helps remediate TBI related symptomology, including circadian disruption. Finally, research should aim to not only include both sexes, as a European cohort that analysed gender differences found women exhibit worse outcomes following TBI (Mikolic et al., 2020), but also take comorbid impairments such as sleep disruption into consideration. This is an extremely important consideration given that many mTBI patients who present with insomnia showed a greater number of circadian rhythm sleep wake disorders (Zalai et al., 2020).

This review article has raised several important questions. First, can we differentiate between symptoms of TBI and those of circadian desynchrony? Secondly, if we can attenuate pathophysiological immune response, or rapidly resynchronize circadian rhythms following TBI, will this aid in recovery? Thirdly, what are the individual differences in outcome following TBI and how are they associated with the degree of circadian desynchrony? In order to answer the first question, we can try to distinguish the contribution of circadian desynchrony to post TBI symptoms by comparing animals placed in a state of chronic circadian desynchronization without a TBI to animals having sustained a TBI on behavioural and molecular measures. It also might be possible to examine clock gene rhythms in various brain and peripheral structures following TBI. In order to answer the second question, pharmacological, means can be employed to halt immune suppression of clock genes. In theory, this might help restore entrainment signals from the SCN to aid in circadian resynchronization following TBI. Alternatively, exercise has been shown to have profound phase resetting effects (Mrosovsky, 1996). However, it currently remains to be seen whether a powerful zeitgeber such as scheduled exercise could be therapeutic in restoring circadian desynchrony following TBI. Finally, individual differences in immune response can be explored. It has been well established that aged animals show a modified immune response and outcome to TBI (Ritzel et al., 2019; Sun et al., 2019a) but it remains to be established how the circadian system interacts with age and injury. It has also been found that there are sex differences in response to injury and neuroinflammation (Doran et al., 2019). Importantly, it has also been demonstrated that there are sex differences in response to circadian desynchrony (Qian et al., 2019). Increasing our understanding of the interaction between sex, age and the circadian system in response to TBI can improve the individualization of intervention strategies and management of treatment paradigms. There are clinical measures of circadian dysfunction such as the sleep regularity index, or salivary melatonin levels (Phillips et al., 2017), that could be assessed in TBI patients and correlated to their outcomes. Circadian interventions such as chronobiotics, chronotherapeutics, or sleep hygiene training could also be used to improve outcomes. The investigation of these important questions, which explore the relationship between the immune system, circadian rhythms, and TBI, have the potential to transform the way we conceptualize future TBI pathology and treatments.

## Credit author statement

Glenn R Yamakawa: Conceptualization, Writing - original draft, Writing - review & editing. Rhys D Brady: Writing - review & editing. Mujun Sun: Writing - review & editing. Stuart J Mcdonald: Writing - review & editing. Sandy R Shultz: Writing - review & editing. Richelle Mychasiuk: Writing - original draft, Writing - review & editing.

## Declaration of competing interest

The authors have no competing interests to declare.
